# Inadvertent Platelet Transfusion from Monkeypox Virus–Infected Donor to Recipient, Thailand, 2023

**DOI:** 10.3201/eid3003.231539

**Published:** 2024-03

**Authors:** Jiratchaya Puenpa, Duangnapa Intharasongkroh, Sompong Vongpunsawad, Dootchai Chaiwanichsiri, Yong Poovorawan

**Affiliations:** Center of Excellence in Clinical Virology, Chulalongkorn University Faculty of Medicine, Bangkok, Thailand (J. Puenpa, S. Vongpunsawad, Y. Poovorawan);; National Blood Center, Thai Red Cross Society, Bangkok (D. Intharasongkroh, D. Chaiwanichsiri);; FRS(T), The Royal Society of Thailand, Sanam Sueapa, Dusit, Bangkok (Y. Poovorawan)

**Keywords:** Mpox, monkeypox, viruses, platelets, transfusion, blood, donation, Thailand, donor

## Abstract

In Thailand, platelet product from a blood donor was transfused to a recipient who had dengue. Two days later, the donor was confirmed to have monkeypox virus infection. Monkeypox virus DNA was undetectable in recipient specimens up to 2 weeks after transfusion. The recipient remained asymptomatic at 4 weeks of monitoring.

Monkeypox virus (MPXV), a double-stranded DNA virus that primarily infects rodents in sub-Saharan Africa, causes mpox disease. MPXV is a member of the genus *Orthopoxvirus* in the family *Poxviridae*. MPXV clade I is endemic to Central Africa and clade II to West Africa. Clade II is further subdivided into IIa and IIb. Strains from the recent global emergence appear to belong to clade IIb (https://nextstrain.org/mpox/all-clades).

The potential to unknowingly transmit MPXV from donated blood products exists despite routine stringent screening of bloodborne pathogens at donation centers. Thailand first reported mpox in a 27-year-old male tourist from Africa in Phuket province on July 21, 2022; nonoutbreak sporadic infections have since been identified (*1*). By May 2023, ≈40 infections had been laboratory-confirmed. Infections surged after Pride Festivals, which took place in Bangkok and Pattaya City in June 2023; infections peaked in August and then declined. As of November 4, 2023, the Ministry of Public Health Thailand (MoPH) had identified 582 infections (563 male and 19 female patients; median age 33 years, age range 1–64 years) and 2 deaths. Here, we describe an unintended administration of platelets from an MPXV-infected donor to a dengue-infected recipient and the subsequent follow-up to monitor for potential MPXV transmission.

On July 24, 2023, an apparently healthy 22-year-old man donated whole blood at the National Blood Center (NBC) of the Thai Red Cross in Bangkok (Figure). That afternoon, he experienced fever and malaise. On July 26, itchy skin rash and lesions appeared on his hands, feet, and anus, which prompted him to go to a hospital. His doctor sought consultation with the Department of Disease Control at MoPH, where samples of the skin lesion, oropharyngeal swab, and plasma were tested for MPXV by real-time PCR to detect the F3L gene region (BioPerfectus, https://www.bioperfectus.com). MPXV DNA was detected only in the lesion (cycle threshold [Ct] 21.7) and oropharyngeal (Ct 31.5) swab samples.

**Figure:**
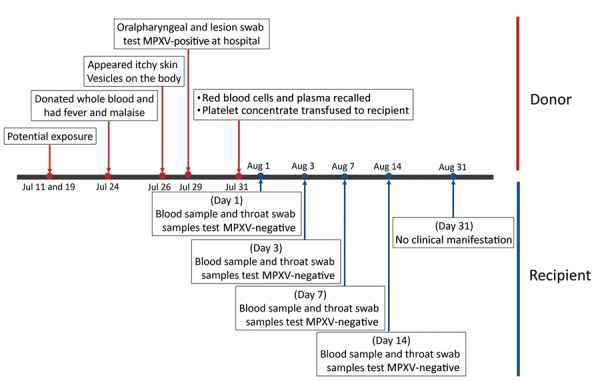
Timeline of MPXV-infected blood donor (red) and platelet recipient (blue), Thailand, 2023. MPXV, monkeypox virus.

NBC processes blood donations individually and routinely screened for hepatitis B/C and syphilis. Derived products from donations are primarily leukocyte-poor red cells, leukocyte-depleted pooled platelet concentrate, and fresh frozen plasma, prepared in accordance with guidelines of the European Directorate for the Quality of Medicines & Healthcare (*2*). Specifically, the platelet concentrate is prepared from a pool of 4 donor buffy coats of the same ABO blood group, diluted with either plasma from one of the buffy coat donations or a platelet additive solution, centrifuged to separate the platelets, filtered to deplete leukocyte, and stored for bacterial testing before distribution.

On July 31, the NBC was alerted to the potential of an MPXV-contaminated donation, which prompted recalls of all blood components derived from the 22-year-old donor. That same day, red blood cells and plasma derived from the donor materials were successfully retrieved and destroyed; however, the platelet concentrate had already been administered to an 11-year-old female recipient who had ongoing dengue infection.

To characterize MPXV in the donation, our laboratory received residual donor plasma and red cells that the NBC had, from which we extracted DNA by using the magLEAD 12 gC instrument (Precision System Science, https://www.pss.co.jp) according to the manufacturer’s instructions. We tested for MPXV DNA by generic real-time PCR to detect the tumor necrosis factor receptor gene located at the terminal inverted repeat region on the MPXV genome, in accordance with the US Centers for Disease Control and Prevention protocol (*3*). We confirmed the result using conventional PCR to amplify the DNA helicase and Schlafen protein genes (Appendix, https://wwwnc.cdc.gov/EID/article/30/3/23-1539-App1.pdf). We Sanger sequenced amplicons, and deposited nucleotides into GenBank (accession nos. OR790439–40).

Plasma yielded detectable MPXV DNA (Ct ≈35); red blood cells did not. Phylogenetic analysis of the DNA helicase gene sequence suggests that the MPXV strain in the donor belonged to clade IIb (lineage B) and genetically clustered with strains previously identified in Taiwan, Japan, and the United States (88% bootstrap support) (Appendix Figure). 

MPXV DNA was undetectable in serum and throat swab samples collected from the platelet recipient on August 1, 3, 7, and 14. No mpox-associated symptoms were evident 4 weeks posttransfusion. Incubation period for mpox is 3–17 days (mean 8.5 days) (*4*,*5*).

We posit that there was a low risk for transfusion-transmitted infection for several reasons. First, detection of MPXV DNA in the residual donated plasma does not indicate infectious virus, as was shown in a viral load study using cell culture as surrogate for infectivity (*6*). Thus, nucleic acid detection does not prove the presence of viable or infectious virus, as Cohen et al. demonstrated in a smallpox-vaccine study (*7*). We pooled and extensively prepared platelet products from multiple donors, which may have diluted out any residual virus before transfusion 1 week later. In conclusion, our study shows that a blood donation from a donor with detectable MPXV viral DNA did not appear to transmit the infection to a pooled-platelet recipient.

Appendix Additional information about platelet transfusion from monkeypox virus–infected donor to recipient, Thailand.
