# Supplementary material for: Inadvertent Platelet Transfusion from Monkeypox Virus–Infected Donor to Recipient, Thailand, 2023
Source: Emerg Infect Dis. 2024 Mar;30(3):603–5. doi: 10.3201/eid3003.231539 (PMC10902518; doi:10.3201/eid3003.231539)
Supplement: Appendix — Additional information about platelet transfusion from monkeypox virus–infected donor to recipient, Thailand. [file 23-1539-Techapp-s1.pdf]

Appendix DOI: http://doi.org/10.3201/eid3003.231539

EID cannot ensure accessibility for supplementary materials supplied by authors. Readers who have difficulty accessing supplementary content should contact the authors for assistance.

Inadvertent Platelet Transfusion from Mpox-Infected Donor to Recipient, Thailand, 2023

Appendix

Gene Amplification and DNA Sequencing

The DNA helicase and Schlafen protein gene sequences were amplified using semi-nested RT-PCR with the primers specified in Appendix Table, resulting in amplicon lengths of 315 bp and 314 bp, respectively. The first-round PCR were performed using PerfectTaq MasterMix PCR system, according to the manufacturer's instructions (5 PRIME, Darmstadt, Germany). The amplification conditions for PCR involved 40 cycles with the following parameters: denaturation at 94 °C for 30 seconds, annealing at 50 °C for 30 seconds, and extension at 68 °C for 1 minute 45 seconds. A final extension step at 68 °C for 5 minutes was included. In the second-round PCR reaction, 1 µL of the first-round reaction served as a template, using second-round primers and PerfectTaq MasterMix (5 PRIME, Darmstadt, Germany) in accordance with the manufacturer's instructions. Subsequently, 40 cycles were conducted with the following conditions: denaturation at 94 °C for 30 seconds, annealing at 50 °C for 30 seconds, and extension at 72 °C for 90 seconds, followed by a final extension at 72 °C for 5 minutes. The sequencing and product amplification were performed simultaneously in both the forward and reverse directions at First BASE Laboratories Sdn Bhd (Selangor Darul Ehsan, Malaysia).

Appendix Table. Primers used for conventional real-time PCR assays

Target region	Primers	Sequence (5´-3´)	Position	Strand
DNA helicase	F129088	CACTCCAGCACCCGCAGAG	129088-129107	Sense
	F129182	ATGTCACTATTAAAGATGGAGTAT	129182-129206	Sense
	R129496	AGTGAAGAGTGATGTATAGAGG	129474-129496	Antisense
Schlafen protein	F167584	TTACTACTGTAGACGTGCATGG	167584-167606	Sense
	F167644	TGCTTCCGATTCCAAATCTGG	167644-167665	Sense
	R167957	AAGACATGCTCCCATAGTCTTC	167935-167957	Antisense

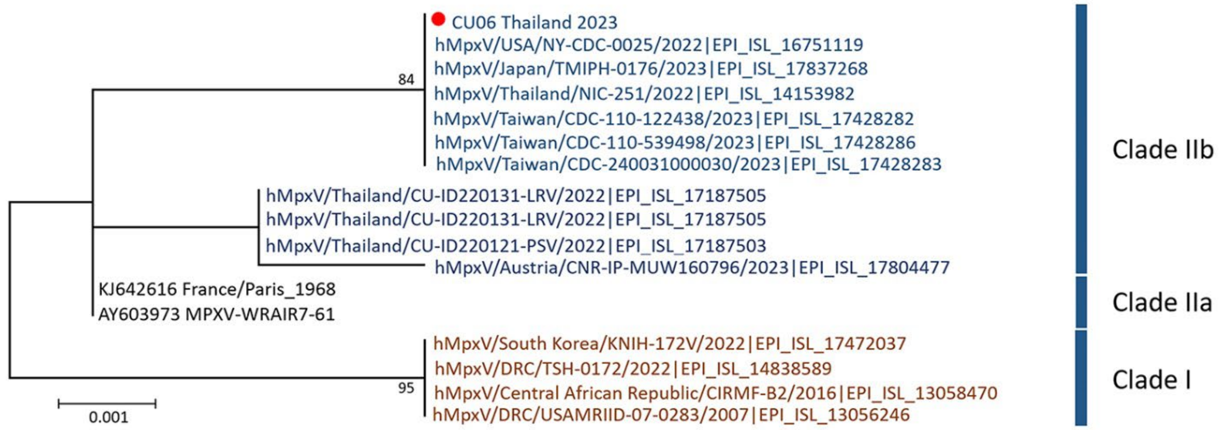

Appendix Figure. Phylogenetic analysis of the DNA helicase gene region (position 123,565–123,898 nt). The phylogenetic tree was constructed using the neighbor-joining method with 1,000 bootstrap replicates and implemented in MEGA version 7 (www.megasoftware.net). Evolutionary distances were computed using the maximum composite likelihood method. Bootstrap values >75 are shown. Scale bar represents substitutions per site.
